# A Comparative Evaluation of Allogenic Cancellous Particulate Chips and Autologous Bone Graft From Symphysis in the Regeneration of Periodontal Intrabony Defect: A Randomized Controlled Clinico-Radiographic Study

**DOI:** 10.7759/cureus.72678

**Published:** 2024-10-30

**Authors:** Manisha Rout, Nitin Tomar, Mayur Kaushik, Shivi Khattri, Roopse Singh

**Affiliations:** 1 Periodontology, Swami Vivekanand Subharti University, Meerut, IND; 2 Periodontology, Lokpriya Hospital, Meerut, IND; 3 Periodontology and Implantology, Subharti Dental college and Hospital, Swami Vivekanand Subharti University, Meerut, IND; 4 Periodontology, Subharti Dental College and Hospital, Swami Vivekanand Subharti University, Meerut, IND

**Keywords:** allograft, alveolar bone loss, autografting, bone, bone replacement materials

## Abstract

Background and objective

Periodontal therapy primarily aims to regenerate periodontal supporting tissues lost due to periodontitis. Autogenous bone grafts (ABG) are viewed as the gold standard method in bone regeneration and they have fewer drawbacks. Hence, many different bone-regenerating materials can be used including allografts, which have excellent biological qualities. This study compares the relative success rates of ABG and mineralized irradiated cancellous bone graft (MICBG) in repairing intraosseous defects caused by periodontal disease.

Methods

The study involved 30 patients presenting with intraosseous defects; 15 each were randomly allocated to two groups after obtaining informed consent: Group A (MICBG) and Group B (ABG). Baseline clinical indicators were evaluated three and six months after surgery. Defect depth (DD) and radiographic bone fill (RBF%) were the two radiographic parameters measured at baseline and six months after surgery.

Results

We observed a statistically significant decrease in radiographic DD between the two groups after six months. However, the mean difference between the two groups for RBF% was statistically non-significant (p>0.05).

Conclusions

Based on our findings, both bone grafts are equally effective in reducing pocket probing depth (PPD). Our results endorse MICBG as a promising alternative to ABG in regenerating periodontal intraosseous defects. The choice of the type of bone graft depends on the decision of the clinician based on the size of the intrabony defect and the availability of the donor site.

## Introduction

The development of intraosseous defects due to periodontal disease is characterized by alveolar bone loss. When bone loss occurs, the primary objective of periodontal therapy is to restore the lost periodontium. The initial treatment for periodontitis involves scaling and root planing (SRP) [1]. However, the mechanical debridement alone is insufficient to regenerate the lost bone. Hence, SRP is supplemented with other biomaterials for periodontal regeneration [2]. These biomaterials include autogenous bone grafts (ABG), demineralized freeze-dried bone allografts (DFDBA), bovine bone xenografts, and synthetic bone substitutes. ABG is associated with an excellent combination of the three biological properties of bone grafts: osteoconduction, osteoinduction, and osteogenesis [3]. Since ABG-containing hematopoietic marrow has the greatest potential for osteogenesis over time, they are considered the gold standard in bone grafting [4].

However, although they have the potential to enhance the production of new bone, several limitations exist in terms of their use. These include higher surgical time and expense, limited supply, and increased patient morbidity/risk of bacterial contamination, which have necessitated the search for alternatives [5]. Allografts, in contrast with many bone replacement grafts, possess both osteoconductive and osteoinductive qualities thanks to growth factors (especially bone morphogenetic proteins) that support three-dimensional tissue ingrowth within their bone scaffold [5,6].

Only a few studies [7,8] have compared ABG with allograft in the literature, and they have yielded inconsistent outcomes. In light of this, it is essential to determine how these grafts differ in terms of their capacity for bone fill. Hence, to provide definitive data, this study aimed to compare and assess the bone fill of ABG from symphysis with that of mineralized irradiated cancellous bone graft (MICBG). The research question we addressed was as follows: "Are allografts as effective as autografts in terms of defect depth (DD) and percentage of radiographic bone fill (RBF%) in patients with intrabony defects?"

## Materials and methods

Study design and registration

**Figure 1:**
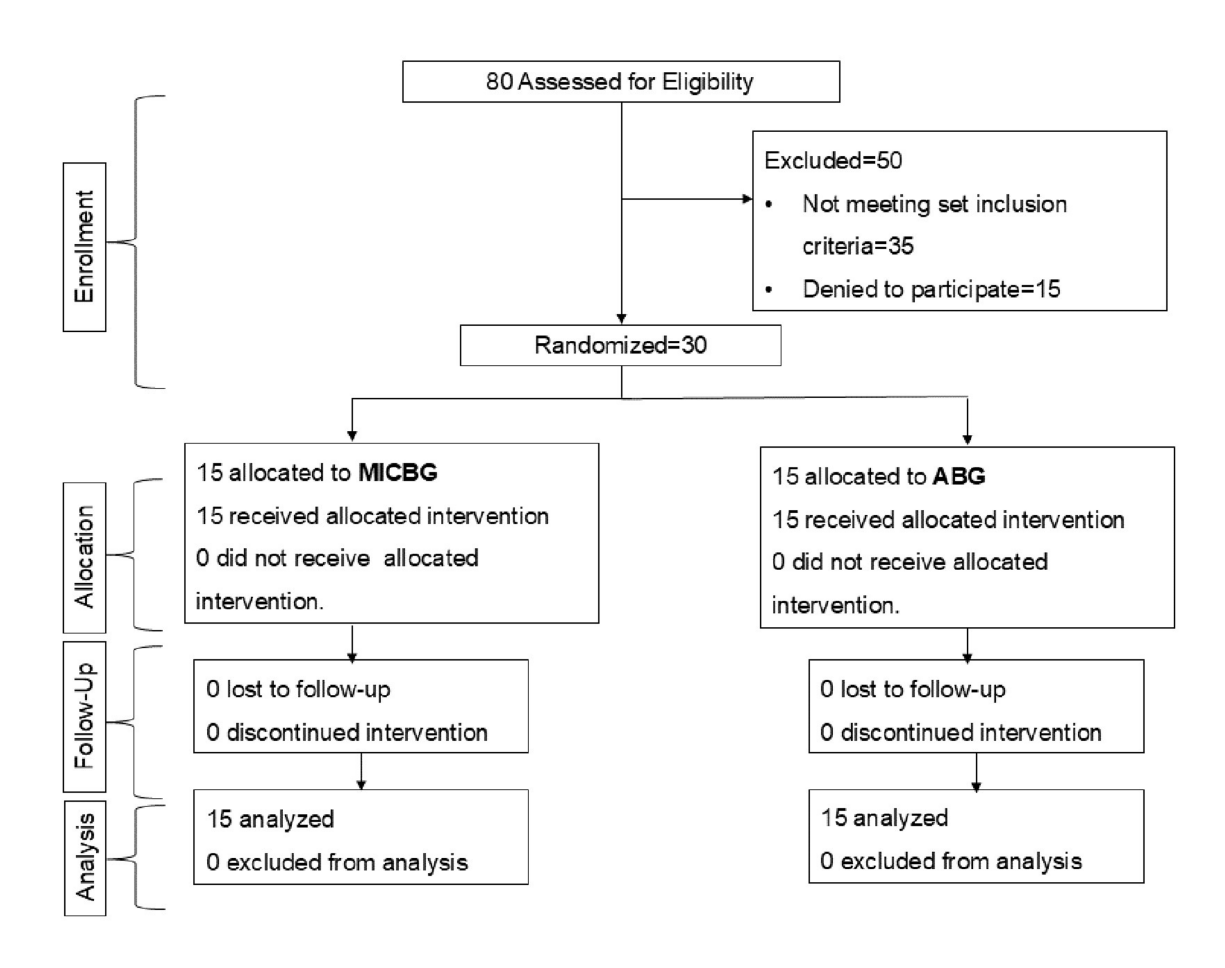
CONSORT flow chart depicting allocations ABG: autogenous bone graft; CONSORT: Consolidated Standards of Reporting Trials; MICBG: mineralized irradiated cancellous bone graft

We conducted a single-blind parallel, randomized controlled trial at Subharti Dental College and Hospital, Swami Vivekanand Subharti University (SVSU). The trial was prospectively registered under the Clinical Trials Registry - India (www.ctri.nic.in) (#CTRI/2023/10/059274) and adhered to the CONSORT statement (http://www.consort-statement.org/) [9]. The CONSORT flowchart is shown in Figure 1. This study was conducted from October 2023 to July 2024.

Sample size calculation

G*Power 3.0.10 was used to compute the sample size. The study's power was set at 80% and the error at 5% (0.05). Thirty patients were randomly divided into two groups. The sample size was calculated from the data obtained from a previous study [7]. 

The sample size formulae used were as follows:

 n = [(σ_1_^2 ^+ σ_2_^2^)/(z_1-α/2 _+ z_1-β_)^2^]/D^2^

The notations for the formulae were as follows:

n = sample size to be determined

σ_1 _= standard deviation of Group 1

σ_2 _= standard deviation of Group 2

D = difference in group means

z_1-α/2_ = two-sided z value (e.g. z = 1.96 for 95% confidence interval).

z_1-β _= power

Substituting the values from the previous study [7],

σ_1_ = standard deviation of Group 1 = 1.49 

σ_2_ = standard deviation of Group 2 = 0.93

D = difference in group means = 1.3

k = ratio = n2/n1 = 1 

z_1-α/2_ = 1.96 at 95% confidence level

z_1-β _= 0.84 at 80% power

Effect size: 1.04 

Substituting these values in above mentioned formula, the total sample size estimated was 15 per group.

Ethical approval and informed consent

The study protocol was approved by the Institutional Review Board of the University Ethics Committee Medical, SVSU (approval no: SMC/UECM/2022/431/229). All procedures followed the ethical standards of the Committee on Human Experimentation (institutional/national) and the Declaration of Helsinki as revised in 2013. All patients participating were informed and educated about the objectives and duration of the study. A written informed consent document was signed by each participant before inclusion in the study.

Inclusion and exclusion criteria

The inclusion criteria were as follows - age group: 20 - 60 years; patients with Stage I/II/III and Grade A/B periodontitis [10]; systemically healthy individuals; and those with pocket probing depth (PPD) ≥5 mm in maxillary and mandibular posterior teeth. The exclusion criteria were as follows - immunocompromised patients; patients who had undergone periodontal surgery in the past six months at the same site; pregnant and lactating females; patients consuming tobacco in any form (smoke/smokeless); and those with cone-beam CT (CBCT) images showing pathologies such as cysts or tumors.

Patient selection and randomization

Thirty patients aged between 30-50 years were recruited from the Outpatient Department of Periodontology. The clinician (SK) was responsible for the enrollment of participants based on the inclusion and exclusion criteria. They were then randomized to Group A (MICBG) and Group B (ABG) by computer-assisted randomizer software. SK assigned the participants to intervention groups. 

Allocation Concealment

In this study, opaque, sealed envelopes with randomly generated treatment assignments were used to accomplish allocation concealment. The envelopes were prepared by an independent team member, and they were opened only after participants were enrolled and consented, ensuring that group assignments remained unpredictable, thereby subsequently preventing selection bias. Because of the study's sample size and six-month follow-up period, no interim analysis was carried out.

Blinding Protocol

In the study, only the clinical examiner (NT) was blinded to treatment assignments (MICBG or ABG) and measuring parameters like PPD and relative attachment level (RAL) at various intervals. This blinding minimized potential bias in outcome assessment related to expectations of the graft types. The operator (MR), responsible for performing the surgeries, was informed of the specific interventions to ensure accurate procedural application, particularly for the preparation of autogenous grafts. This setup was both logistically feasible and scientifically sound, as it maintained the integrity of the surgical processes while allowing for unbiased assessments.

The clinical examiner (NT) was calibrated at different time points. The intraclass correlation coefficient for Gingival Index (GI), Plaque Index (PI), PPD, RAL, and DD was calculated for assessment of intraexaminer reliability by using the "kappa test". It was found to be moderate to excellent (Table 1).

**Table 1:** Assessment of intraexaminer reliability DD: defect depth; GI: Gingival Index; PI: Plaque Index; PPD: pocket probing depth; RAL: relative attachment level

Variable	Intraclass correlation coefficient	Interpretation
GI	1.000	Excellent agreementGI
PI	1.000	Excellent agreement
PPD	0.73	Moderate agreement
RAL	0.89	Good agreement
DD	1.000	Excellent agreement

Outcomes measures

Primary Outcome

The primary outcomes included DD (mm) and RBF%. With the aid of radiovisiography (RVG) and the long cone paralleling technique, intraoral periapical radiographs were obtained. DD was measured as the distance from the cementoenamel junction (CEJ) to the bottom of the bony defect and BD is the distance from the alveolar crest of the adjacent tooth to the depth of the bony defect [11].

To calculate RBF%

Bone defect area (BDA) was calculated by the formula:

BDA = ½ *Defect Depth*BD 

RBF% after six months was then calculated using the formula:

RBF(%) = BDA at baseline - BDA at six months/BDA at baseline x 100

All the measurements were carried out by a single examiner (NT) throughout the study and he was unaware of the surgical procedure carried out

Secondary Outcome

The clinical parameters that were recorded pre-surgically and post-surgically at intervals of three months and six months were as follows: RAL measured using occlusal stent made from cold cure acrylic (measured from the lower border of the stent to the base of the pocket) [12], PPD, PI [13], and GI [14].

Non-surgical Phase

Before surgery, complete supragingival and subgingival SRP were done for every patient. Oral hygiene instructions were given to all the patients. Patients were recalled after one week for re-evaluation. Once the patients exhibited good oral hygiene, they were randomized into one of the groups for surgical intervention. 

Surgical procedure

**Figure 2:**
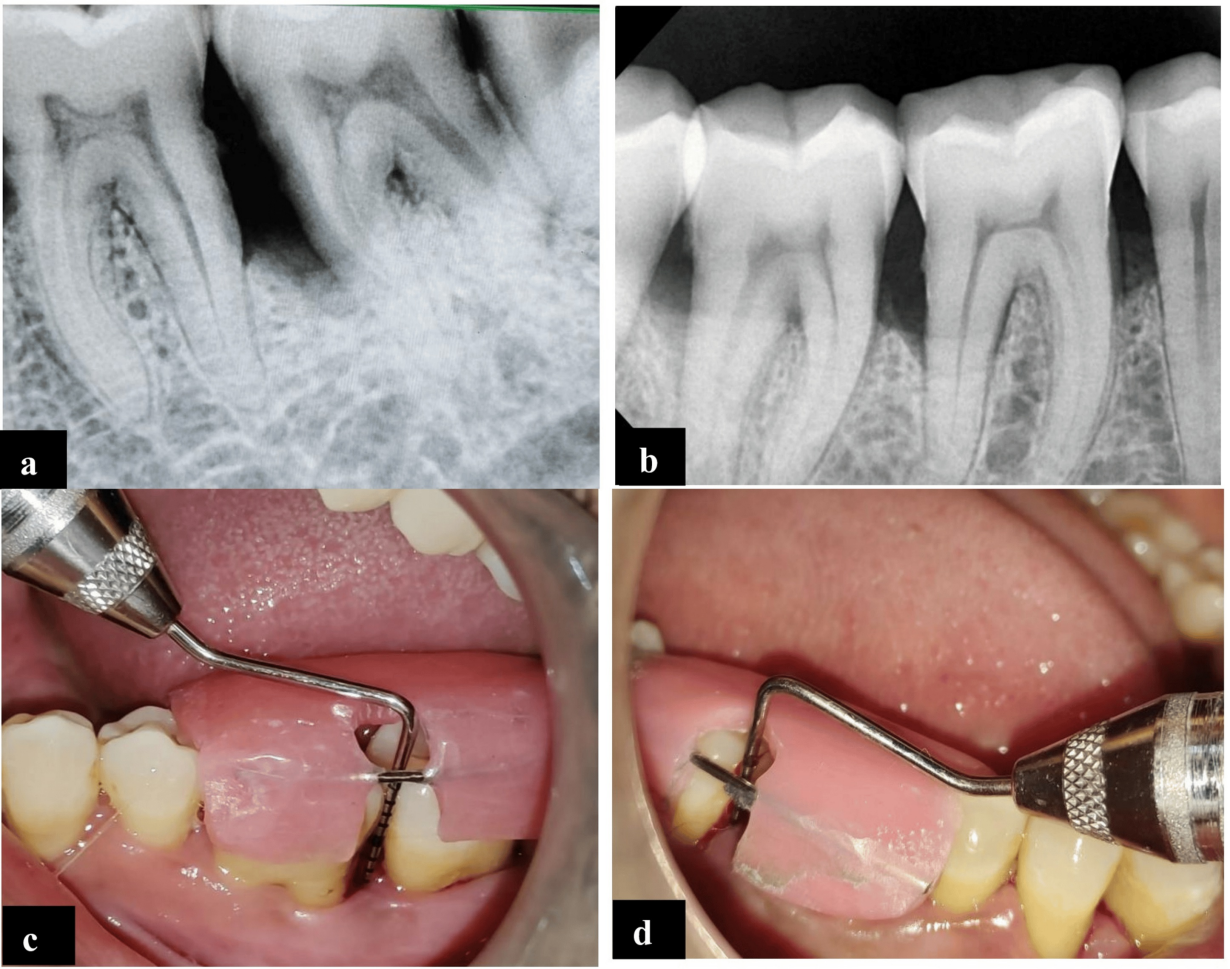
(a) Group A preoperative RVG irt 37 (MICBG). (b) Group B preoperative RVG irt 46 (ABG). (c) Preoperative RAL irt 37. (d) Preoperative RAL irt 46 ABG: autogenous bone graft; irt 37: in relation to tooth number 37; irt 46: in relation to tooth number 46; MICBG: mineralized irradiated cancellous bone graft; RAL: relative attachment level; RVG: radiovisiography

The day of the surgery was considered the baseline. Intraoral asepsis was performed with 0.2% chlorhexidine gluconate rinse and extraoral sepsis was done with povidone iodine solution. Preoperative RVG and RAL measurements were carried out for both groups (Figures 2a-2d). Local Anesthesia was administered (4% articaine + epinephrine 1:100,000) before carrying out surgery.

Procurement for ABG (Group B)

**Figure 3:**
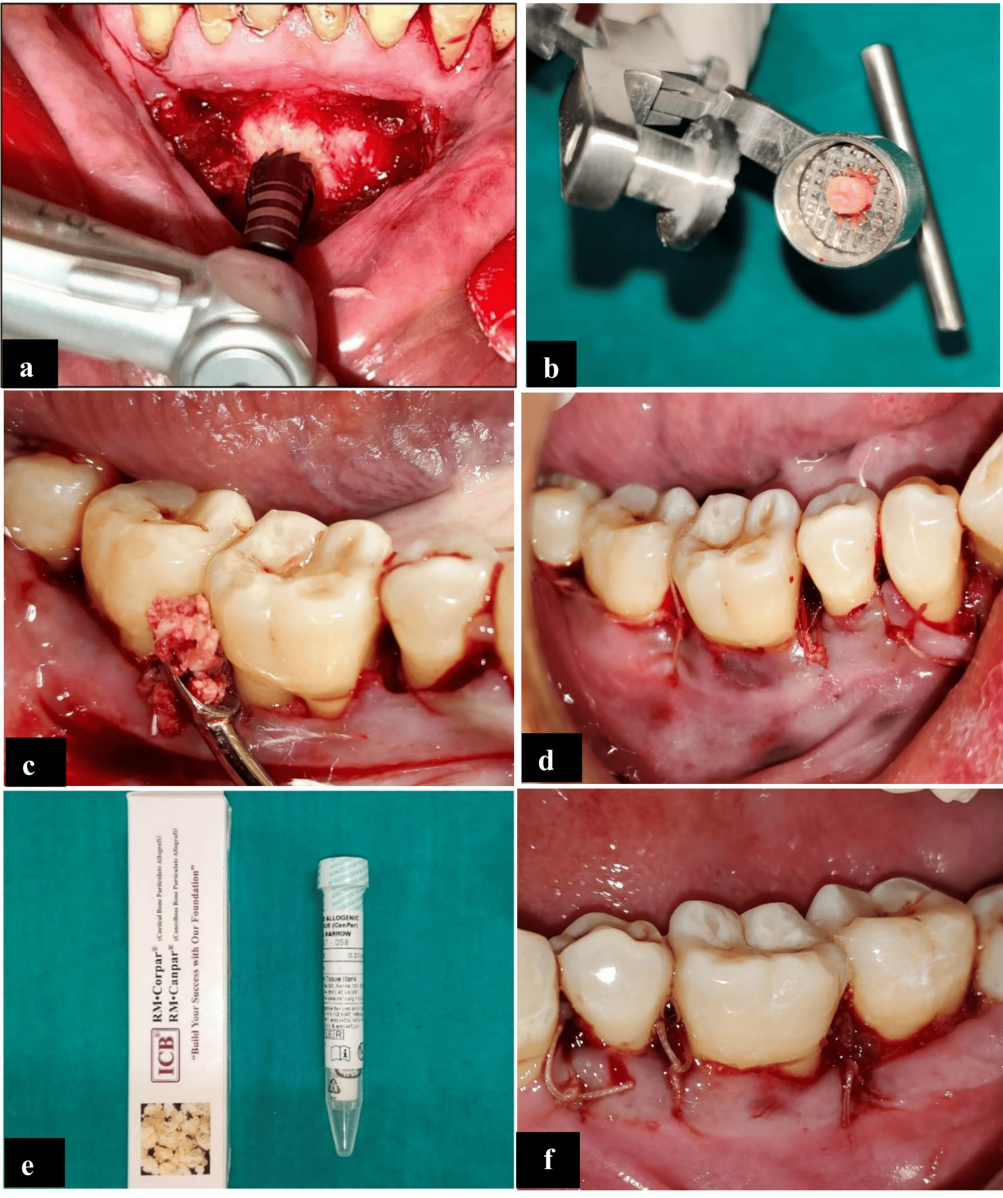
(a) ABG harvested using 6 mm trephine bur. (b) ABG transferred to bone mill and crushed. (c) Autograft condensed at defect site irt 46. (d) 3-0 resorbable sutures placed; (e) 0.5cc MICBG allograft utilized irt 37 (f) 3-0 resorbable sutures placed ABG: autogenous bone graft; irt 37: in relation to tooth number 37; irt 46: in relation to tooth number 46

In Group B, preoperative CBCT was taken before harvesting. The ABG was harvested from the symphyseal region using a 6 mm trephine bur, maintaining the 8 mm distance from root apices of teeth, distance from the border of the mandible, and mental foramen [15] (Figure 3a). Once the cortico-cancellous block graft was harvested it was crushed into particulate bone chips using a bone mill (Figure 3b).

Management of Intrabony Defect Site

A crevicular incision was made and a full-thickness mucoperiosteal flap was raised. The area was meticulously degranulated using a curette. Pre-suturing was done to avoid spillage of the graft material. The bone graft (ABG) was condensed using a bone graft condenser (Figure 3c) in Group B. A complete approximation of the flap was done using a 3-0 resorbable suture (Figure 3d). A similar surgical protocol was followed in Group A (MICBG) (Figures 3e, 3f).

Postoperative management

**Figure 4:**
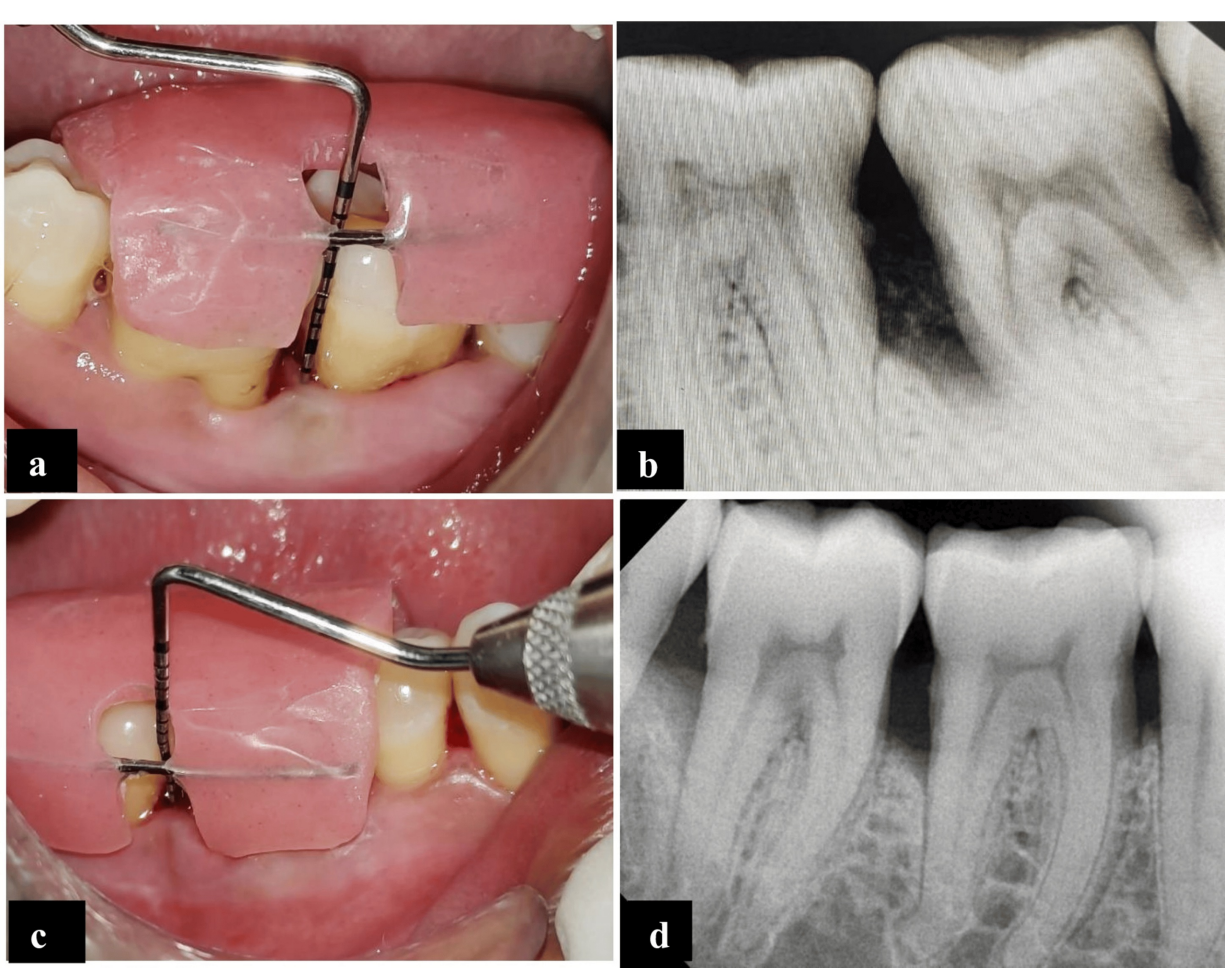
(a) Postoperative RAL after 6 months irt 37. (b) Postoperative RVG after 6 months irt 37 (MICBG). (c) Postoperative RAL after 6 months irt 46. (d) Postoperative RVG irt 46 after 6 months (ABG) ABG: autogenous bone graft; irt 37: in relation to tooth number 37; irt 46: in relation to tooth number 46; MICBG: mineralized irradiated cancellous bone graft; RAL: relative attachment level; RVG: radiovisiography

Analgesics and antibiotics (500 mg amoxicillin, TDS; 400 mg metronidazole, TDS; and 800 mg ibuprofen, TDS) were provided for five days, along with postoperative instructions. A mouthwash containing povidone-iodine (2% w/v) was prescribed to the subjects for two weeks following surgery. The clinical parameters were recorded at baseline as well as three months and six months postoperatively. The radiographic parameters were recorded at baseline and after six months (Figures 4a-4d).

Statistical analysis

The SPSS Statistics software (IBM Corp., Armonk, NY) was used to tabulate and statistically analyze all data on clinical and radiographic parameters. Mean and standard deviations (SD) were calculated for continuous measures (mean DD, RBF%, PI, GI, PPD, and RAL). The independent student t-test and Mann-Whitney U test were used for the intergroup analysis of PI, GI, PPD, RAL, mean DD, and RBF%. Wilcoxon signed-rank test was used to measure the intragroup analysis between mean DD. A significance threshold of p<0.05 was set. 

The data were assessed for normality by using the Shapiro-Wilk test. The data of GI, PDD, RAL, and RBF% were distributed normally, whereas the data of DD and PI did not follow a normal distribution. Therefore, a comparison of DD and PI between the two groups was performed using the Mann-Whitney U test. A comparison of paired data of DD was done using the Wilcoxon signed-rank test.

## Results

A total of 80 patients were assessed for eligibility. Among them, 35 did not meet the inclusion criteria and 15 declined to participate as they could not comply with all recall visits. Hence, 30 patients, 15 in each group, were randomized and given the specified treatments. All patients completed the follow-up visits and compiled with the study recall appointments (Figure 1). Patients’ baseline clinical characteristics are shown in Table 2. No significant differences were observed between the two groups in terms of baseline clinical parameters (p>0.05). After three and six months of follow-up, significant improvements in soft tissue parameters (PI and GI) were observed in both treatment groups compared to preoperative measurements (Table 2). Both groups had a non-significant difference (PI and GI) at each time interval, indicating that all study participants maintained appropriate oral hygiene. As ABGs involve the second surgical site, the patients had to be well informed about the potential post-operative complications, including edema, and the healing of the donor site. The patients were kept on recall period after 24 hours and at one week and two weeks to re-evaluate for potential complications.

**Table 2:** Intergroup comparison between the two groups ^*^P<0.05 (statistically significant) Intergroup comparison between the two groups for clinical parameters PI, GI, PPD, and RAL at baseline and at 3 and 6 months by independent student t-test. Intergroup comparison for mean DD and RBF% between two groups at baseline and 6 months by Mann-Whitney U test Group A: patients treated with MICBG (RM-CanPar®). Group B: patients treated with ABG ABG: autogenous bone graft; DD: defect depth; GI: Gingival Index; MICBG: mineralized irradiated cancellous bone graft; PI: Plaque Index; PPD: pocket probing depth; RBF%: radiographic bone fill percentage; RAL: relative attachment level; SD: standard deviation

Clinical parameters	Group A (mean ± SD)			Group B (mean ± SD)						
	Baseline (mm)	3 months postoperatively (mm)	6 months postoperatively (mm)	Baseline (mm)	3 months postoperatively (mm)	6 months postoperatively (mm)	P-valueat baseline	P-valueat 3 months	P-valueat 6 months	
PI	1.12 ± 0.29	0.74 ± 0.31	0.36 ± 0.31	0.97 ± 0.18	0.59 ± 0.14	0.25 ± 0.12	0.217	0.25	0.233	
GI	1.13 ± 0.30	0.71 ± 0.30	0.43 ± 0.09	1.19 ± 0.35	0.77 ± 0.37	0.54 ± 0.37	0.605	0.655	0.283	
PPD	8.13 ± 1.13	4.73 ± 0.70	4.07 ± 1.03	8.00 ± 1.41	4.53 ± 0.83	2.87 ± 0.74	0.777	0.484	0.001^*^	
RAL	12.00 ± 1.31	8.60 ± 0.74	7.93 ± 1.39	11.93 ± 1.49	8.40 ± 0.99	6.80 ± 1.27	0.897	0.534	0.027^*^	
										DD	0.84 ± 0.50	-	0.47 ± 0.46	0.97 ± 0.50	-	0.56 ± 0.35	0.367	-	0.041^*^	
RBF%	-	-	45.01 ± 26		-	33.28 ± 17.07	-	-	0.165	

We observed a reduction in PPD and gain in RAL from baseline to six months within both groups, which were statistically significant (p<0.05) (Table 2). Radiographically, the mean change in DD and mesiodistal width after six months was statistically highly significant between the two groups (p≤0.05). The mean DD reduction at six months for Group A was 0.47 ± 0.46 whereas it was 0.56 ± 0.35 for Group B, indicating that DD in Group B (ABG) was significantly greater than that in Group A (MICBG) (Table 2). Also, after six months, the intragroup analysis for the mean DD in each group demonstrated significantly lower values compared to the baseline (p=0.001) (Table 3). The RBF% for Group A and Group B was 45.01 ± 26.90 and 33.28 ± 17.07 respectively, indicating that the difference between both groups was statistically non-significant (p=0.165) (Table 2).

**Table 3:** Intragroup comparison of mean DD at baseline and 6 months using the Wilcoxon signed-rank test ^*^P<0.05 (statistically significant) Group A: patients treated with MICBG (RM-CanPar®). Group B: patients treated with ABG ABG: autogenous bone graft; DD: defect depth; MICBG: mineralized irradiated cancellous bone graft; SD: standard deviation

Parameter	Group	Baseline	At 6 months postoperatively	Difference	P-value
DD, mean ± SD	Group A	0.84 ± 0.50	0.47 ± 0.46	0.37	0.001^*^
	Group B	0.97 ± 0.50	0.56 ± 0.35	0.41	0.001^*^

## Discussion

A wide array of bone substitutes are regularly used to promote bone formation and periodontal regeneration. Particulate cancellous chips and autografts have long been considered the "gold standard" for bone grafting techniques, offering a plentiful supply of bone and marrow cells with osteogenic potential. It has been reported that bone grafts obtained using a bone mill and bone scraper exhibited more cell viability than bone drilling techniques and piezo surgery [16]. A randomized controlled clinical experiment assessing the outcomes of different harvesting methods on bone formation and graft resorption in vivo[17] found that bone graft treated in a bone mill was linked to more newly created bone. Therefore, in the current clinical study, ABG was obtained using a trephine bur from the symphysis region, and the bone graft was then crushed using a bone mill. The ABG from symphysis was relatively easy to obtain. It is more effective as it increases the surface area when the graft is crushed using the bone mill.

The cortico-cancellous nature of ABG provides a structurally sound osteoconductive medium with osteoinductive and osteogenic support for filling intraosseous defects. However, the requirement for donor material warrants a second surgical site, which has various drawbacks - the infrequent availability of intraoral donor sites, risk of bacterial contamination, and limited quantity of bone grafts were some of the manacles of ABG - which made it difficult to treat larger defect sites [18]. Clinical studies [19,20] have established that treating intraosseous periodontal deformities with a combination of guided tissue regeneration (GTR) and ABG did not yield additional benefits. The current trial employed ABG only without the use of extra biologics, as studies have shown a significant improvement with both materials.

Various bone graft substitutes are currently available, which include allografts, xenografts, and alloplasts. Among these, allografts have better osteoconductive and osteoinductive properties. It has been demonstrated in studies [21,22] that the freeze-dried bone allograft (DFDBA) group exhibited a remarkable improvement in bone density when compared to DFDBA. Also, the histological analysis revealed that after 18 to 20 weeks of recovery, FDBA produced almost twice as much vital bone as it did after only 8-10 weeks. MICBG has been utilized in the present study. A hydrated version of MICBG with superior mechanical handling properties is available. It condenses readily at the site of a defect because of its cohesive nature.

In the present study, the clinical parameters did not show a statistically significant difference from baseline to six months postoperatively between the two groups. This indicated that both groups showed similar outcomes regarding soft tissue parameters. The RBF% in Group A (MICBG) and Group B (ABG) showed a notable increase at the six-month follow-up. There were statistically non-significant differences between the two groups based on the mean difference between them, which was found to be 11.73%. Participants in the current trial showed improvement over baseline at different time intervals for all clinical parameters in both groups. This aligned with various studies [23,24,25] that showed that combining DFDBA plus OFD resulted in a higher level of clinical improvement than OFD alone. From baseline to six months, PPD and RAL significantly improved for both groups. The reduction in DD after six months between the two groups was almost similar. At six months postoperatively, Group B (ABG) had a substantially greater reduction in mean DD (p<0.05) when compared to Group A (MICBG). From the baseline period to six months postoperatively, the mean DD reduction between the groups revealed a significant difference (p=0.001) (Table 2). 

Limitations

This study has a few limitations. A larger sample size and longer-term analysis might yield more significant results, especially regarding operating time for ABG harvesting and donor site morbidity linked to ABG. Treating bigger intraosseous defect sites was challenging due to the inadequate amount of available ABG. Furthermore, no histologic investigations were performed to ascertain the kind of bone fill. Future studies could employ modern imaging techniques like CBCT for volumetric analysis and accurate bone level measurement. 

ABG is cost-effective and easily available. As the allograft used in this study was imported from Rocky Mountain Tissue Bank, the cost of bone graft was higher because of the import duties. However, the usage of allograft exempts the patients from involving a second surgical site. Hence, both bone grafts can be used depending on the patient's choice and the availability of the type of graft.

## Conclusions

The goals of periodontal regeneration using a bone graft constitute reversing the disease process, reducing probing depth, gaining clinical attachment, healing lost bone, and histologic reconstruction of the attachment apparatus - which comprises new bone, cementum, and periodontal ligament - in an area previously exposed to bacterial plaque. After six months, both groups in our study demonstrated a significant reduction in DD. The p-values provide strong evidence that the reason for this decrease in DD is not random variation, but rather the treatment or intervention that was implemented. Group A had a substantially lower mean DD than Group B during the six months. ABG is linked to a higher reduction in DD than MICBG due to the rapid revascularization that occurs around graft particles because of its osteogenic impact. In contrast, thanks to its osteoconductive capability, MICBG has also demonstrated a favorable level of bone regeneration. Given the challenges associated with ABG, allogenic bone (MICBG) obtained from another individual and frequently processed by tissue banks offers a great substitute for ABG.

## Figures and Tables

**Figure 5:**
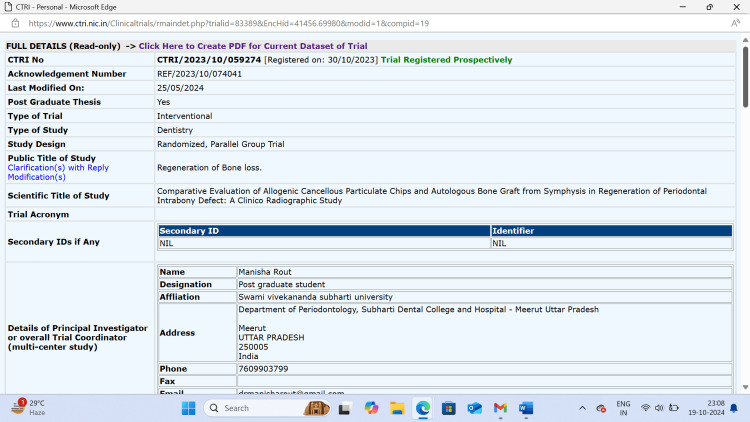
Screenshot 1

**Figure 6:**
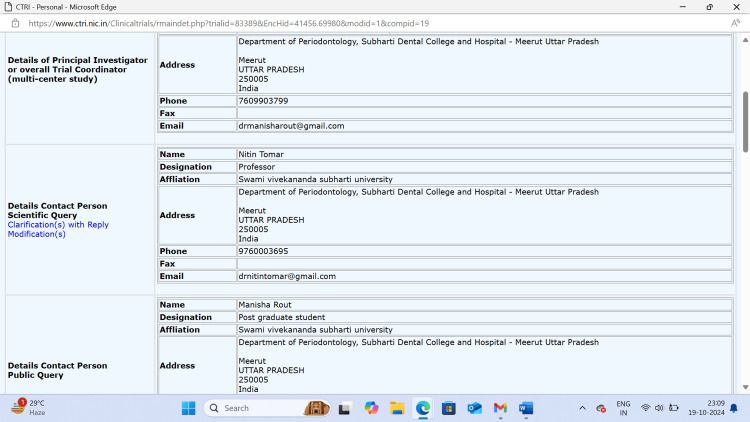
Screenshot 2

**Figure 7:**
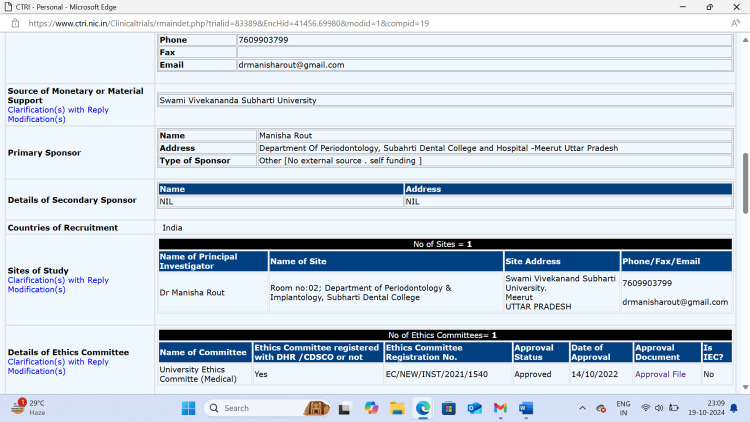
Screenshot 3

**Figure 8:**
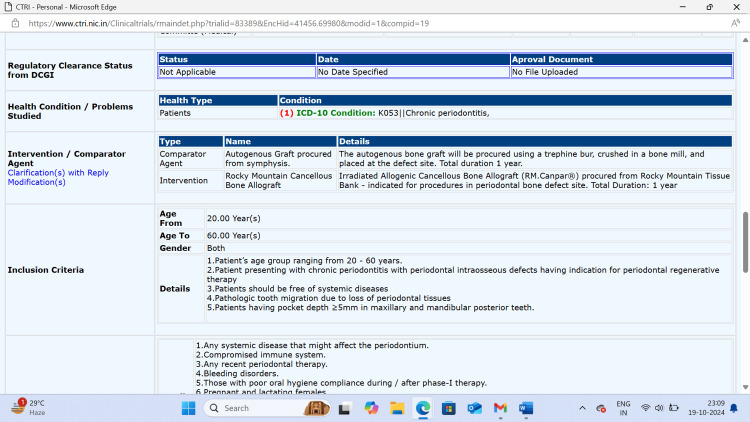
Screenshot 4

**Figure 9:**
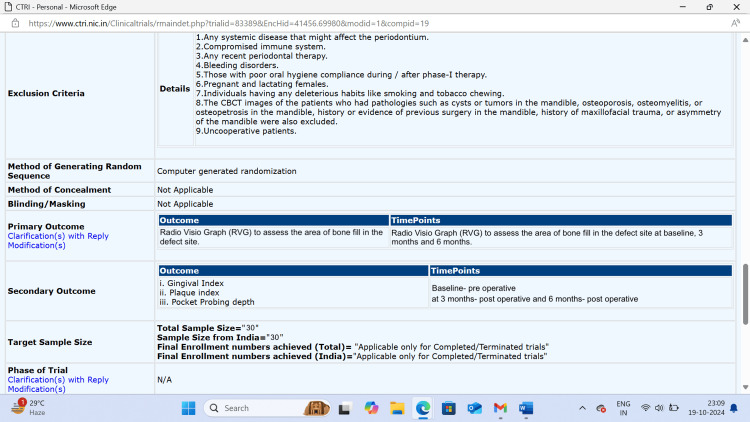
Screenshot 5

**Figure 10:**
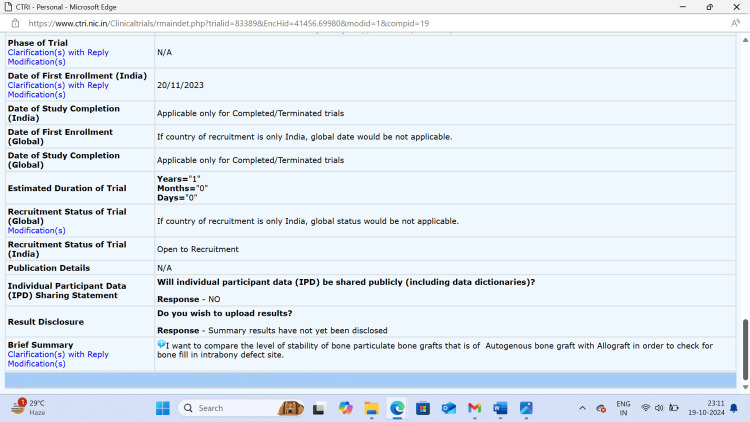
Screenshot 6

**Figure 11:**
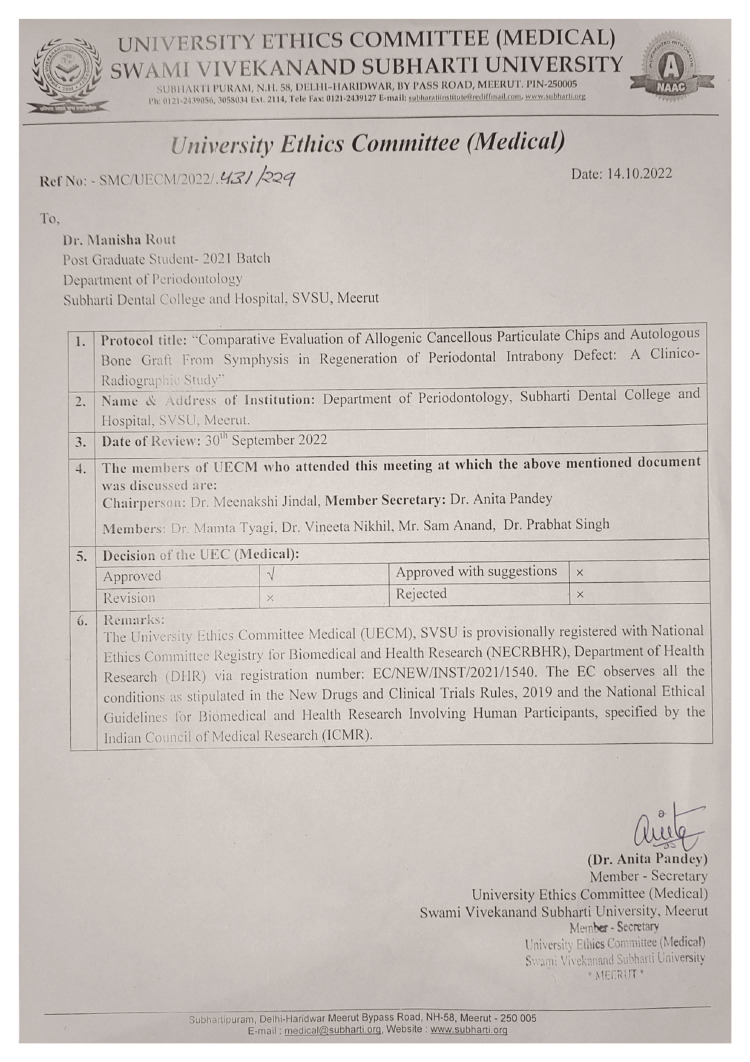
IRB approval letter

## Appendices

Screenshots of the Clinical Trials Registry - India (CTRI) registration page are presented in Figures 5-10.

The IRB approval letter is shown in Figure 11.
